# Control of Human African Trypanosomiasis in the Nola‐Bilolo Focus, Central African Republic, 2020–2024: Outcome of Capacity Building, Improved Diagnosis and Treatment

**DOI:** 10.1155/japr/8625795

**Published:** 2026-03-08

**Authors:** Pierre-Marie Douzima, Mireille Cornelia Ingrid Denissio Morissi Nalingbo, Yvon Andjingbopou, Veerle Lejon, Romaric Nzoumbou-Boko

**Affiliations:** ^1^ National Programme to Control Human African Trypanosomiasis, Ministry of Health, Bangui, Central African Republic, behdasht.gov.ir; ^2^ Laboratoire de Parasitologie, Institut Pasteur de Bangui, Bangui, Central African Republic, pasteur-bangui.org; ^3^ Faculté des Sciences de la Santé, Université de Bangui, Bangui, Central African Republic; ^4^ Institut de Recherche pour le Développement, UMR INTERTRYP, Université de Montpellier-IRD-CIRAD, Montpellier, France, ird.fr

**Keywords:** diagnosis, fexinidazole, human African trypanosomiasis, mAECT, Nola-Bilolo, treatment

## Abstract

This study is aimed at evaluating the inclusion of miniature anion‐exchange centrifugation technique (mAECT) in the diagnosis and fexinidazole as treatment of gHAT in Central African Republic (CAR) after capacity building. A cross‐sectional pilot study was conducted during a mass diagnostic campaign from 16 September to 22 October 2022 in Nola‐Bilolo, a historic focus in CAR. The serological test, card agglutination test for trypanosomiasis (CATT), was performed on whole blood and diluted plasma to screen participants, followed by a confirmatory parasitological test by capillary tube centrifugation (CTC) and mAECT. Positive cases were treated mainly with fexinidazole. A total of 2070 participants were screened, out of an estimated population of 3584, representing a participation rate of 58%. The seroprevalence of HAT was 1.6% (CI 95%: 77%–99%) (33/2070) by the CATT plasma end‐dilution titre ≥ 1:16. Blood from serological subjects was examined microscopically after concentration by CTC and mAECT was positive for trypanosomes in 48.48% (CI 95%: 31%–66%) (16/33) and 87.88% (CI 95%: 77%–99%) (29/33) of cases, respectively. The prevalence of microscopically confirmed HAT was 0.77% (CI 95%: 0.40%–1.15%) and 1.4% (CI 95%: 1%–2%) by CTC and mAECT, respectively. Twenty‐one (72.41%) patients were treated with fexinidazole with a 0% interruption rate. The introduction of fexinidazole (2021) and mAECT (2022) likely contributed to the rise in reported cases (from 45 in 2021 to 111 in 2024) and improved screening coverage in the study area, reflecting enhanced case detection and access to care. There is a necessity to establish diagnostic quality assurance and to reinforce the other control measures including vector control.

## 1. Background

The World Health Organization (WHO) has set the goal of eliminating human African trypanosomiasis (HAT) as a public health problem by 2020, and to stop its transmission by 2030 [1]. HAT is caused by the vectorial transmission of a protozoan parasite, *Trypanosoma brucei gambiense* in West and Central Africa, and *T. b. rhodesiense* in East Africa [2]. It puts 70 million people at risk throughout Sub‐Saharan Africa, and it is usually fatal if untreated or inadequately treated [3]. The HAT involving *T. b. gambiense* evolves two distinct successive phases determining its two pathological stages; the haemolymphatic Stage I, characterised by intermittent fever, intense headache, insomnia, painless lymphadenopathy, anaemia, local oedema and rash, this stage, also known as the nonsevere phase, may be asymptomatic or subclinical. The meningoencephalitis Stage II is distinguished by cachexia, somnolence and signs of central nervous system involvement [4].

Although HAT has been eliminated as a public health problem in Togo, Côte d’Ivoire, Benin, Uganda, Equatorial Guinea, Ghana, Rwanda, Guinea, Chad for gambiense HAT and in Rwanda and Kenya for rhodesiense HAT, it remains a public health problem in Angola, Central African Republic (CAR), Congo Republic, Democratic Republic of Congo (DRC) and South Sudan. This elimination includes the integration of control activities through surveillance in endemic areas [5]. Despite significant progress in diagnostic techniques, treatment tools and vector control, the disease remains endemic in CAR [6].

The CAR remains the second country to have reported the most cases of HAT after the DRC, with 111 cases and 303 cases, respectively, for a total of 546 cases reported in Sub‐Saharan Africa in 2024 [7]. However, these registered cases are not representative of endemic foci, which are often inaccessible, mainly due to insecurity. Conflict and war are recognised as determining factors in the risk of spreading infectious diseases. The incidence of sleeping sickness and epidemics in CAR is largely underestimated. Moreover, HAT cases have been reported in non‐endemic areas such as Bangassou.

The mainstay of intervention strategies has been combined diagnosis and treatment of infection together with vector control. Several diagnostic strategies have been used, including passive, active or reactionary approaches by the serological and parasitological techniques through microscopic observation of blood or other biological fluids [3]. Treatment of HAT is aimed not only at curing the patient but also at interrupting the transmission chain, and it is therefore important to detect infected individuals as early as possible. The control of HAT due to *T. b. gambiense* (gHAT) is compromised by low sensitivity of routine parasitological tests [8]. A screening algorithm that is both highly sensitive and highly specific is essential for the detection and treatment of patients. There are two important complementary approaches to control and eliminate the disease. The country′s elimination programme was grounded in the WHO pillars for HAT control: case detection (by active and passive screening, then confirmation) and treatment, vector control, surveillance and supportive partnerships [9]. In CAR, the Card Agglutination Test for Trypanosomiasis (CATT) coupled with blood examination by microscopy was applied since the creation of the national trypanosomiasis control programme (PNLTHA) in 1995. For the PNLTHA, plasma with end‐dilution titre ≥ 1:16 is considered a serological suspect and selected for the parasitological test after concentration in the capillary tube centrifugation (CTC). However, the yield of CTC is low and another parasitological concentration technique, the mini anion exchange centrifugation technique (mAECT), is 10 times more efficient than CTC, and was introduced. mAECT is recommended as the most sensitive test for demonstrating trypanosomes in human blood and is routinely used in certain endemic countries [10]. mAECT is considered the reference technique for the detection of trypanosomes but is more difficult to implement because it requires qualified human resources, logistics and is more expensive.

From 2021, fexinidazole has been introduced as a treatment for HAT ,and in 2022, parasitological testing using the mAECT has been introduced and tested in one focus of the CAR.

In this paper, we present the first result of a screening campaign in a focus in CAR including mAECT in the diagnostic for gHAT and fexinidazole as treatment.

## 2. Materials and Methods

### 2.1. Study Design, Site and Capacity Building

In November 2020, health workers have been trained in the use of fexinidazole (cascade training) by the PNLTHA in collaboration with HAT Platform support by Drugs for Neglected Diseases initiative (DNDi). In October 2021, the PNLTHA in collaboration with WHO organised a five‐day HAT diagnostic refresher training to build competencies and skills in HAT diagnosis, especially in serological testing (CATT and RDT) and concentration methods (CTC and mAECT) coupled with microscopy. The training took place in the parasitology laboratory of the Pasteur Institute of Bangui. After these training courses, the study was conducted in the Nola province of the CAR. Nola‐Bilolo is one of the four foci of CAR, the most historic and is still active, and it can be investigated by PNLTHA because of its relative safety. Active enrolment of the target population was carried out from 16 September to 22 October 2022 by one mobile team of PNLTHA supported by local technicians, using routine diagnostic protocols as prescribed by the PNLTHA, and the new treatment protocol was applied.

### 2.2. Assessment of Coverage Rate

The coverage rate is defined as the ratio between the number of people who underwent CATT screening during our study period and the target population (number of people in the study area) based on ICASESS census data [11].

### 2.3. Screening and Serological Test

The serological test by the CATT is a direct plate agglutination; it consists in bringing together antigens of trypanosomes, consisting of whole and lyophilized *T. b. gambiense* and the whole blood of the person to be examined. The video component of CATT demonstration can be found at https://www.youtube.com/watch?v=dN56HA6oH2I. The diagnostic algorithm prescribed by the PNLTHA for CAR based on standard protocols described by Magnus et al. [12] Capillary blood was used for performing CATT on whole blood. Quantitation was performed on all positive CATT whole blood samples. It consisted to the successive dilutions (1:2, 1:4, 1: 8, 1:16, and 1:32) of plasma from whole blood. The macroscopic agglutination patterns, ranging from negative to strongly positive, are scored by matching with reference photographs. Serologically suspected individuals with a CATT‐plasma end‐dilution titre ≥ 1:16.

### 2.4. Parasitological Test Confirmation

From all included suspects, 5 ml of venous blood was collected on heparin for sampling for the following parasitological tests: Capillary Centrifugation Technique test, as described by Woo, with four capillaries filled with 60 *μ*L [13]. To maintain quality, slides were prepared, viewed and double‐checked by two professional microscopists under ×40 magnification to search for trypanosomes. PNLTHA parasitological testing protocol uses only the CTC for confirmation.

In mAECT, trypanosomes are separated from 350 *μ*L of whole blood by anion exchange chromatography on diethylaminoethyl cellulose (DEAE) as described by Büscher et al. [14]. Eluted trypanosomes are then concentrated by low‐speed centrifugation, followed by direct microscopic examination of the sediment in a transparent collector tube double‐checked by two professional microscopists under ×40 magnification to search for trypanosomes. The video component of mAECT demonstration can be found at https://www.youtube.com/watch?v=EVQY_5JDPvM.

### 2.5. Treatment

Fexinidazole is a new oral treatment for Stage 1 and nonsevere Stage 2 HAT. General health was qualified as good, altered or bad by the investigators according to their overview of interrogation, physical and neurological exam, vital signs and laboratory results. This treatment does not require hospital admission or a lumbar puncture for all patients, which is likely to ease access for patients. Fexinidazole, an all‐oral medication requiring a 10‐day regimen, participants were enrolled if they were Aged 6 years or older, weighed 20 kg or more and had parasitological confirmation. Patients not eligible for fexinidazole were treated with pentamidine.

### 2.6. Data Management

Baseline sociodemographic characteristics were registered. The data were entered into an Excel 2010 database, and then subjected to standard descriptive statistics (mean, frequency, etc.). To assess the relative sensitivity of the parasitological tests, we compared the number of positives for each test in the number of populations examined using McNemar′s test with *p* values of < 0.05 considered to indicate statistical significance [15].

## 3. Results

### 3.1. General Characteristics of Target Population and Coverage Rate

**Figure 1:**
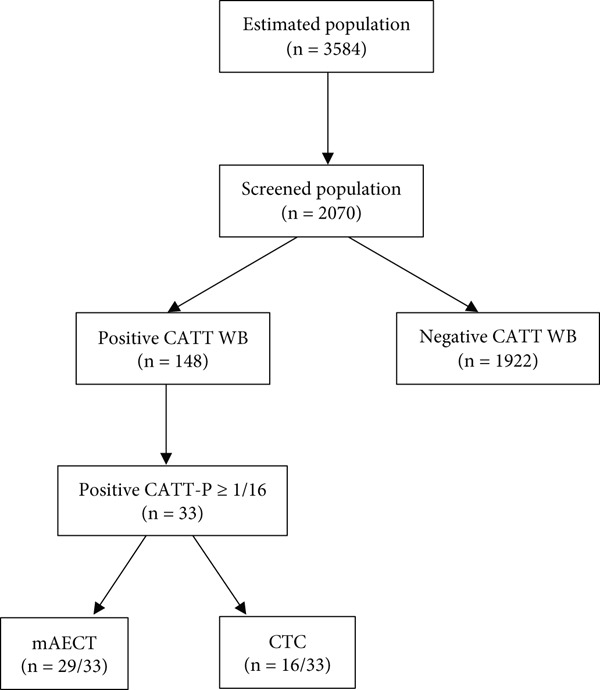
Flowchart of diagnosis design of study.

A total of 2070 participants were enrolled in this study, out of an estimated population of 3584, representing a participation rate of 58% (Figure 1). The age varied between 3 and 67, with an average of 36 years. The participant male–female gender ratio was 0.49.

### 3.2. Serological Results

The undiluted blood CATT screening showed a prevalence of 7.15% (148/2070). The number of positive results for CATT‐plasma diluted at titres of 1:2, 1:4, 1:8, 1:16 and ≥ 1:32 was 48, 45, 22, 16 and 17, respectively (Table 1). In accordance with the PNLTHA diagnostic algorithm for CATT, the 22.30% (33/148) positives at a titre ≥ 1/16, who were serological suspect cases, represent a HAT seroprevalence of (33/2070) 1.6% (CI 95%: 1.09%–2.09%).

**Table 1:** Number of participants according to their titre in CATT.

**CATT—Plasma titre**	**Number of positives**
1:2	48
1:4	45
1:8	22
1:16	16
1:32	17

### 3.3. Parasitological Results

We found that (29/33) 87.88% (CI 95%: 77%–99%) of these subjects were subsequently confirmed by microscopy after concentration by mAECT versus (16/33) 48.48% (CI 95%: 31%–66%) by microscopy after concentration by CTC (*p* *v* *a* *l* *u* *e* < 0.001) (Table 2). The prevalence of parasitologically confirmed HAT was 1.4% (CI 95%: 1%–2%) and 0.77% (CI 95%: 0.40%–1.15%) for mAECT and CTC, respectively. The clinical examination of serological suspects showed that around 94% were asymptomatic and subclinical, and the remaining 6% reported headaches and intermittent fever. There have been no severe cases recorded according to perceptible clinical signs. All mAECT positive patients were treated with oral fexinidazole. Patient receiving treatment referred to Bilolo primary health centre for follow up. The four mAECT negative individuals were placed under periodic medical supervision, and their samples were sent to a specialist laboratory for trypanolysis, and the results were negative.

**Table 2:** Test results of parasitological diagnosis. The number of participants with positive and negative results for each test.

		**mAECT**			**p**
		Positive (*n*)	Negative (*n*)	Total	< 0.001
**CTC**	Positive (*n*)	16	0	16	
	Negative (*n*)	13	4	17	
	Total	29	4	33	

### 3.4. Therapeutical Results

In this study, almost all of the 29 patients diagnosed are in good general health, with a few showing fever. Thus, 21 (72.41%) patients were treated with fexinidazole and eight (27.59%) patients were not eligible for fexinidazole (five patients with a bodyweight less than 20 kg and three patients who do not have the possibility to eat food concomitant with fexinidazole) were treated with pentamidine. Patients were followed up during the treatment for 2 weeks; no adverse events were reported, and no patients discontinued the treatment (0% interruption rate). The assessment of treatment outcome has not been carried out, because it requires follow up for up to 24 months with clinical assessment and laboratory exams.

## 4. Discussion

This study has shown that the Nola‐Bilolo focus is still active. Although this historical focus is the only one of the four in the CAR where surveys for diagnosis and treatment are carried out regularly, the parasite is still circulating. The coverage rate obtained in this study is higher than that of a previous study in the same area estimated at 44% in 2015 [16]. Although screening coverage is acceptable, the 58% participation rate underscores the challenge of eliminating the disease solely through active screening; a large portion of the population (42%) did not undergo active screening, leaving many potential cases undetected, highlighting the need for complementary strategies, including passive screening. It is demonstrated that control and surveillance of HAT should be based on the combined use of active and passive screening in the primary health centres [17]. Although our findings confirm the essential role of active screening and improved tools for a rapid reduction in prevalence in the Nola‐Bilolo focus, achieving the 2030 elimination goals depends on sustainable efforts, particularly via effective passive screening. Previous operational research, such as the work by Mulenga et al. [17], in DRC has demonstrated the successful integration of HAT diagnostic and surveillance activities, supported by new tools, into primary health care facilities. This integration represents the crucial next step for the CAR to consolidate the gains made during mass campaigns. In addition, Chad′s success illustrates the power of the combination of vector control measures, which may have interrupted transmission, medical detection and treatment have played a major role in reducing HAT incidence and elimination [18]. Another successful model of vector control in Ivory Coast with the use of tiny targets to control the tsetse fly, in addition to other vector control approaches, provides sustainable tsetse control to achieve the interruption of *T. b. gambiense* transmission [19]. Published modelling work exemplifies that vector population control between 60% and 90% is sufficient to interrupt transmission to humans; thus, demonstrating the value of adding vector control to screening and treatment efforts [20, 21]. A study, one of the few on the entomological situation in the Nola‐Bilolo focus, carried out in 1991, showed that *Glossina fuscipes fuscipes* and *Glossina palpalis palpalis* were joint vectors of sleeping sickness in the focus of Nola‐Bilolo in the CAR [22]. Recently, the continental atlas of the distribution of tsetse flies in Africa documented that the genus *Glossina* circulates in approximately three‐quarters of the country [23]. Thus, vector control must be adopted and implemented as an important tool in CAR′s national strategy to eliminate gHAT. Although the study took place during the COVID pandemic, we found that COVID did not have a negative impact on the coverage rate. This increase in the coverage rate is thought to be due to the considerable reduction in the use of lumbar puncture following the introduction of fexinidazole. No lumbar punctures were performed during our survey. It has been shown that, in patients without neurologic symptoms and signs, the likelihood of a severe meningoencephalitis stage is deemed low, obviating the need for a lumbar puncture to guide treatment decisions using fexinidazole [24]. Various studies have already highlighted the various barriers that dissuade people from taking part in active gHAT screening and treatment, including lumbar puncture [25]. Another advantage of fexinidazole is that it is an oral formulation, which avoids intramuscular injections and can be administered also to nonhospitalized patients [5]. The coverage rate of our study is also a little higher than that of an active screening study carried out in Boffa (Guinea), estimated at 50.9% and in the high‐endemicity health zone of Kwamouth in the DRC, estimated at 55% [26, 27].

Increasing the rate of curing coupled with improving the diagnostic algorithm is a necessary approach to the control and elimination of HAT in the focus studied. The introduction for the first time of the mAECT in the HAT diagnostic algorithm has improved diagnostic performance. This study has shown that mAECT microscopy is estimated to be two times more sensitive than CTC microscopy. Our values are slightly higher than those in a study in the East Kasai province of the DRC, where mAECT had a performance of 1.3 times more sensitive than CTC [27]. Our results are almost identical to those of a study carried out in the Bandundu Province (DRC) in 2011, where the performance of mAECT was estimated as two times more sensitive than CTC [28]. The prevalence observed appears to be higher than that of a study carried out in the same site 6 years earlier, representing an increase from 1.1% to 1.4% [16]. This increase in prevalence is thought to be due to the introduction of the mAECT, which is more effective than the CTC technique used in 2016. It is documented that CTC has a sensitivity of 500 trypanosomes per mL, whereas mAECT has a sensitivity of 10 to 50 parasites per mL [28]. The prevalence value demonstrated in this study is higher than any documented prevalence of CAR. The diagnostic techniques previously used in CAR certainly underestimated the prevalence of HAT. It is shown that the combination of rapid antibody screening and microscopy‐based diagnosis by mAECT has had a tremendous impact on HAT control over the last decade [29]. Based on this, it would also be better to change the diagnostic algorithm and to perform mAECT when CATT whole blood is positive. Since doing the CATT dilution and doing parasitology only for the 1:16 onwards, it might miss cases due to the higher sensitivity of CATT and safer drugs like Fexinidazole. In a study of systematic review, it is reported that the sensitivity and specificity of CATT on whole blood was higher than the CATT dilution; in addition, the preparation of sample dilutions requires trained personnel and some extra equipment [30]. Other studies have shown that the sensitivity of CATT on whole blood is better, whereas specificity is low compared with CATT dilution [31, 32]. For a screening test, sensitivity is often prioritized over specificity to catch as many cases as possible, even if it means more false positives [33].

The introduction of fexinidazole in 2021 and mini–Anion Exchange Centrifugation Technique (mAECT) in 2022 may partly explain the observed increase in reported cases—from 45 in 2021 to 111 in 2024—as well as the improved screening coverage in the study focus. These innovations likely enhanced case detection and expanded access to diagnosis and treatment [7, 34]. These values may be an underestimation due to low screening coverage rates. These new cases would represent the “tip of the iceberg”, whereas most actual infections represent the much larger, submerged part of the iceberg. In addition, new cases appearing in areas where a disease is not normally found could indicate the emergence of new, undetected “silent foci” of the disease. This exploratory study is therefore intended as a pilot study, based on which a more rigorous study can be planned to update the HAT data in RCA and produce a new map of this disease.

The mAECT must be implemented in the Batangafo, Haut‐Mbomou and Lobaye areas, not forgetting the Mbomou prefecture, which has been a hot spot for over 100 years and has provided sporadic cases recently, because the biomedical technicians of this prefecture are also trained and equipped to allow routine passive detection.

Experience has shown that in countries that have been declared HAT free, the achievement is attributed to robust control and surveillance measures, active and passive or reactive screening of people at risk and targeted vector control, which helps to decrease the number of cases in areas of transmission. Treatment of infected people meant that the vector, the tsetse fly, could no longer transmit the disease to others. Although therapeutic efficacy has not been evaluated, we noticed good therapeutic compliance with fexinidazole. This study indicates that access to quality diagnostic to where people at the greatest risk of infection live promotes identification of cases in earlier stages of infection and improves treatment.

## 5. Limitations

Some limitations are inherent to the set‐up of the study. At the time of the study, we should also be using RDTs, which are available at the PNLTHA in addition to mAECT. In addition, the definition of serological suspect should evolve to include CATT‐plasma end‐dilution titre ≥ 1:8 or without dilution as carried out in certain endemic countries. It would also be better to change the diagnostic algorithm and to perform mAECT when CATT whole blood is positive. The assessment of treatment outcome by the follow up with clinical assessment, laboratory exams and pharmacovigilance would have provided more data enabling the optimization and adaptation of different control strategies in the dynamic of portfolio approach.

## 6. Conclusion

Introducing the mAECT offers an improved diagnostic sensitivity compared with CTC, and in addition, it has been demonstrated that there is still an active focus, with more cases than expected, due to an underestimation where CTC had previously been used. The introduction of mAECT and fexinidazole revealed more new cases. The benefits of using CATT on whole blood for active and passive screening are evident in the scientific literature, the CATT on whole blood could be considered as the most cost‐effective option for use in routine screening for HAT in the CAR. The assessment of treatment effectiveness would have been remarkable because few real‐life studies on the efficacy of fexinidazole treatment were published. Unfortunately, gHAT control in the CAR has received little attention, this contrasting with the global health approach required for elimination. There is an urgent need to update the mapping of gHAT foci in CAR using active and passive screening approaches, and to reinforce control measures including vector control.

## Ethics Statement

The study protocol was accepted by trypanosomiasis experts from the PNLTHA by research authorisation N°024/MSP/DIRCAB/DGELM/DLMTNMNT/SLMTN/PNLTHA.22. The study′s plan was conducted in accordance with the guidelines and requirements of the Ministry of Health; therefore, this was a public health control programme and did not require ethics committee approval. The participants were assured of the confidentiality and anonymity of their data; all being stored securely and only used for research purposes.

## Conflicts of Interest

The authors declare no conflicts of interest.

## Author Contributions

Pierre‐Marie Douzima: methodology, data curation, formal analysis, funding acquisition, investigation, writing—original draft, writing—review and editing. Mireille Cornelia Ingrid Denissio Morissi Nalingbo: methodology, formal analysis, investigation, writing—review and editing. Yvon Andjingbopou: formal analysis, investigation, writing—review and editing. Veerle Lejon: methodology and writing—review and editing.

Romaric Nzoumbou‐Boko: conceptualization, methodology, supervision writing—review and editing.

## Funding

This study was supported by the World Health Organization (10.13039/100004423).

## Data Availability

The data supporting this study′s findings are available from the corresponding author upon request.
